# The Original Rutherford Morison Incision: A Case Report

**DOI:** 10.7759/cureus.52803

**Published:** 2024-01-23

**Authors:** Robyn Cabral, Vijay Naraynsingh

**Affiliations:** 1 Department of General Surgery, Port of Spain General Hospital, Port of Spain, TTO; 2 Department of Clinical Surgical Sciences, The University of the West Indies, St. Augustine, St. Augustine, TTO; 3 Department of Surgery, Medical Associates Hospital, St. Joseph, TTO

**Keywords:** hemicolectomy, rutherford morison incision and hemicolectomy, divarication of rectus abdominis, port site hernia, incisional hernia, diastasis recti, rutherford morison incision, original use

## Abstract

In recent years, the Rutherford Morison incision has become synonymous with renal transplant surgery. However, this incision was originally intended for access to the sigmoid colon and pelvis, particularly in the case of a midline previously scarred from operation. We present a case of a middle-aged female with a caecal tumour, requiring resection. Upon examination, this patient was found to have large concomitant diastasis of the recti. A right-sided Rutherford Morison incision was utilized in performing a right hemicolectomy. Although the advantages of a minimally invasive approach to colonic resections are well described, laparoscopy was not utilized in the case discussed. Due to the wide area of anterior abdominal wall laxity, herniation is likely to develop at both port placement and specimen delivery sites. A similar outcome would result from a midline incision. However, a paramedian is an acceptable alternative to a Rutherford Morison incision in a case like this, as it is known to have very low rates of post-operative incisional herniation. While in modern times, its use may have become repurposed, the Rutherford Morison incision is one which should be remembered and used in the surgeons’ armamentarium to improve clinical outcomes when necessary.

## Introduction

The use of the Rutherford Morison incision has changed over the last few decades. Originally, Professor James Rutherford Morison utilised a curvilinear oblique, left-sided incision, to facilitate access to the sigmoid colon when performing sigmoid colectomies [1]. In more recent years, we have moved away from the application of this incision to use in colorectal surgery, and it has become synonymous with renal transplantation. Herein we describe a case of a right hemi-colectomy, performed via a right-sided Rutherford Morison incision.

## Case presentation

**Figure 1:**
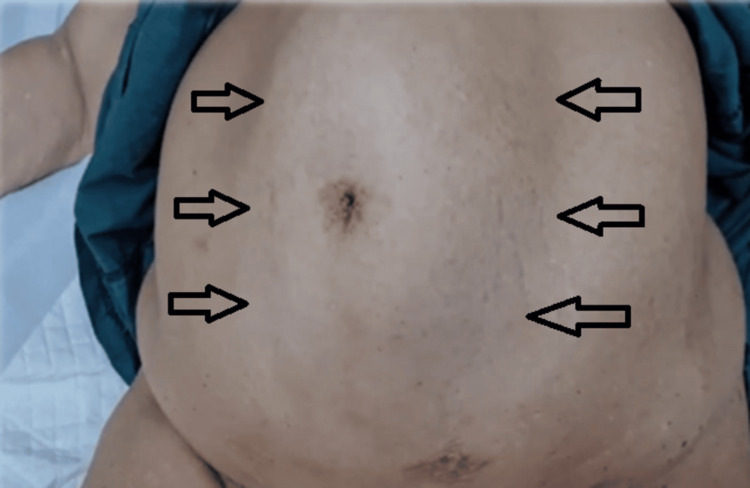
Diastasis of the rectus abdominis evident upon increased intra-abdominal pressure in the supine position.

**Figure 2:**
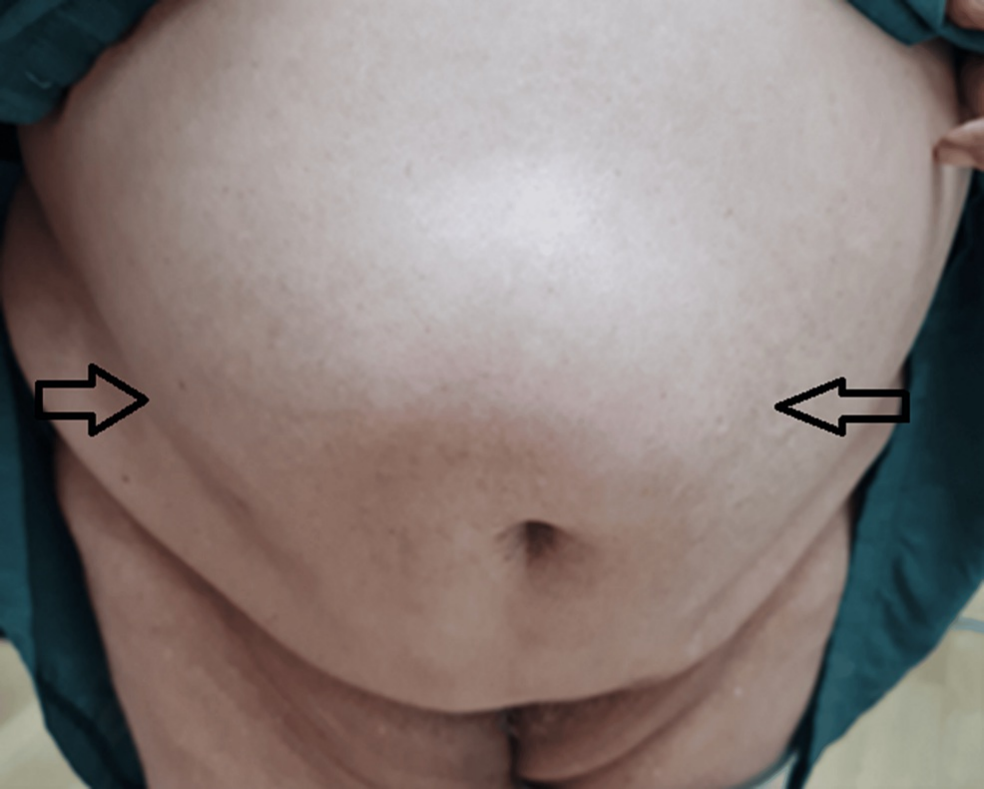
Extensive diastasis of the rectus abdominis seen when standing.

A 60-year-old female presented with isolated complaints of anaemia and weight loss. Save for obesity, this patient was previously well. Upon examination, abdominal palpation was challenging due to increased abdominal girth. However, a large concomitant diastasis of the rectus abdominis (Figures 1, 2) was noted which, according to the European Hernia Society guidelines on the management of rectus diastasis, was classed as T1D3H0 [2]. Blood investigations performed were significant only for a microcytic anaemia, with a normal carcinoembryonic antigen.

**Figure 3:**
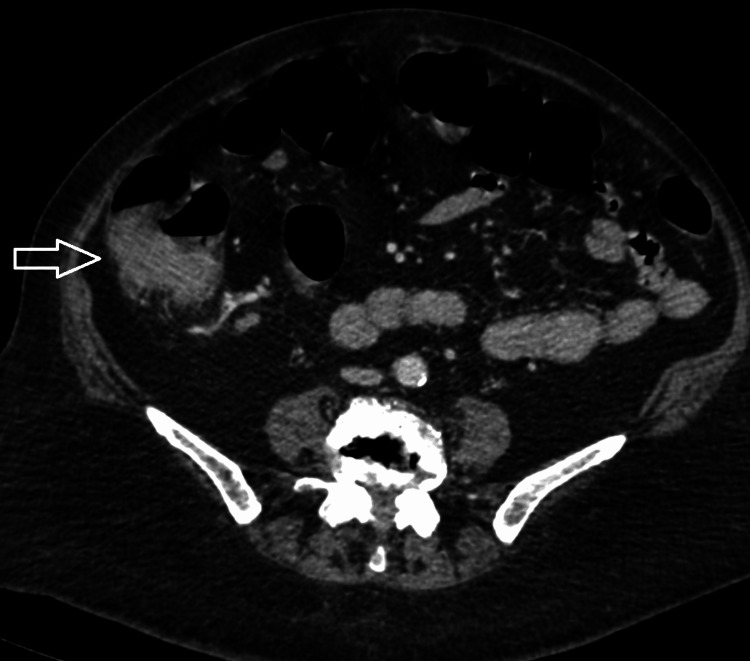
Axial view of caecal mass on computed tomography

**Figure 4:**
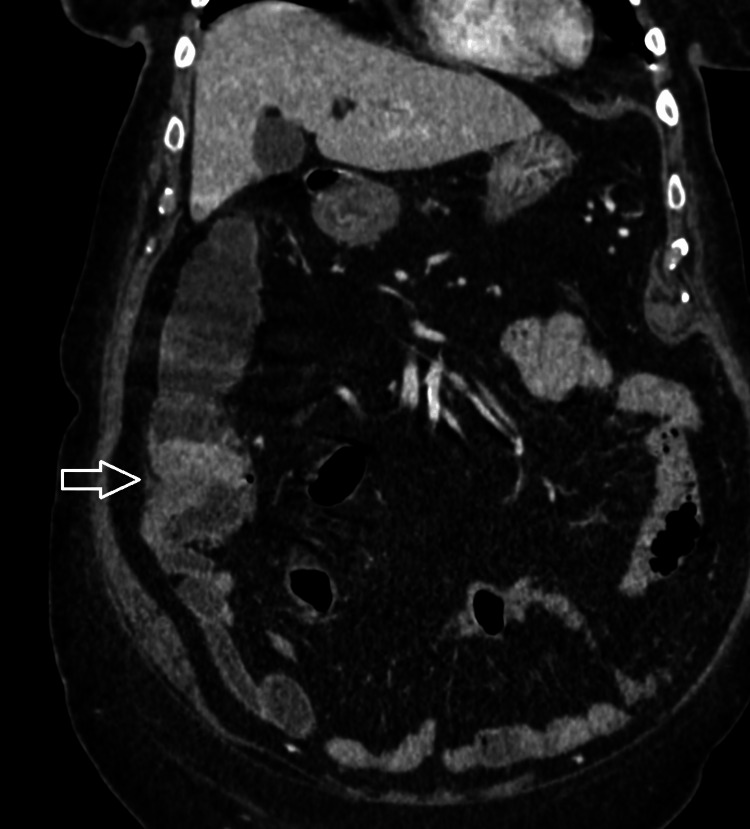
Sagittal view of caecal mass on computed tomography

Radiological imaging revealed a mass present within the caecum (Figures 3, 4) with no distant spread noted. Histological evaluation of biopsy done via endoscopy diagnosed the mass as a caecal adenocarcinoma, and the patient was prepared for operative intervention.

**Figure 5:**
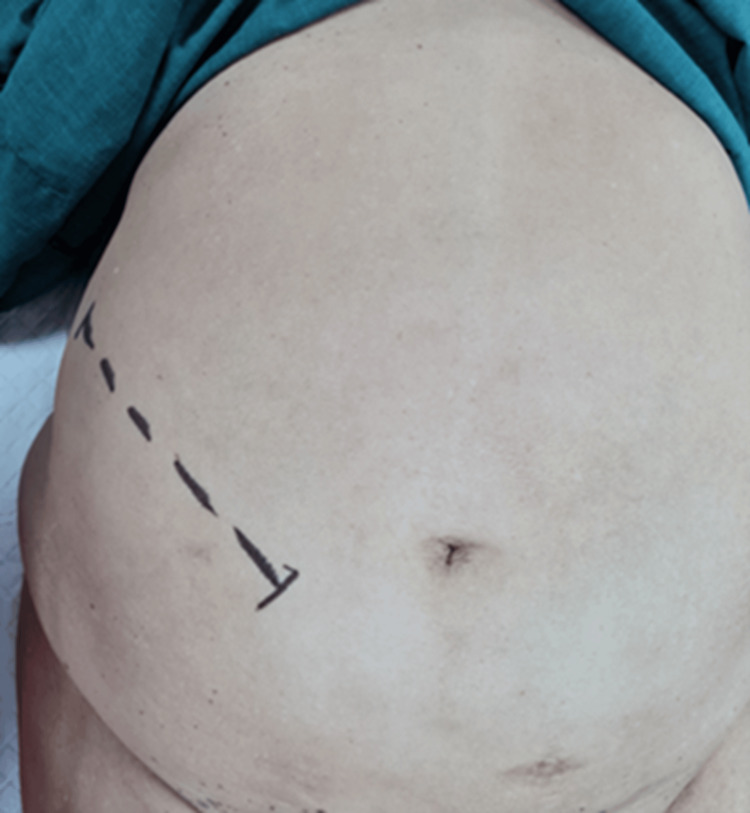
Planned right-sided Rutherford Morison incision

**Figure 6:**
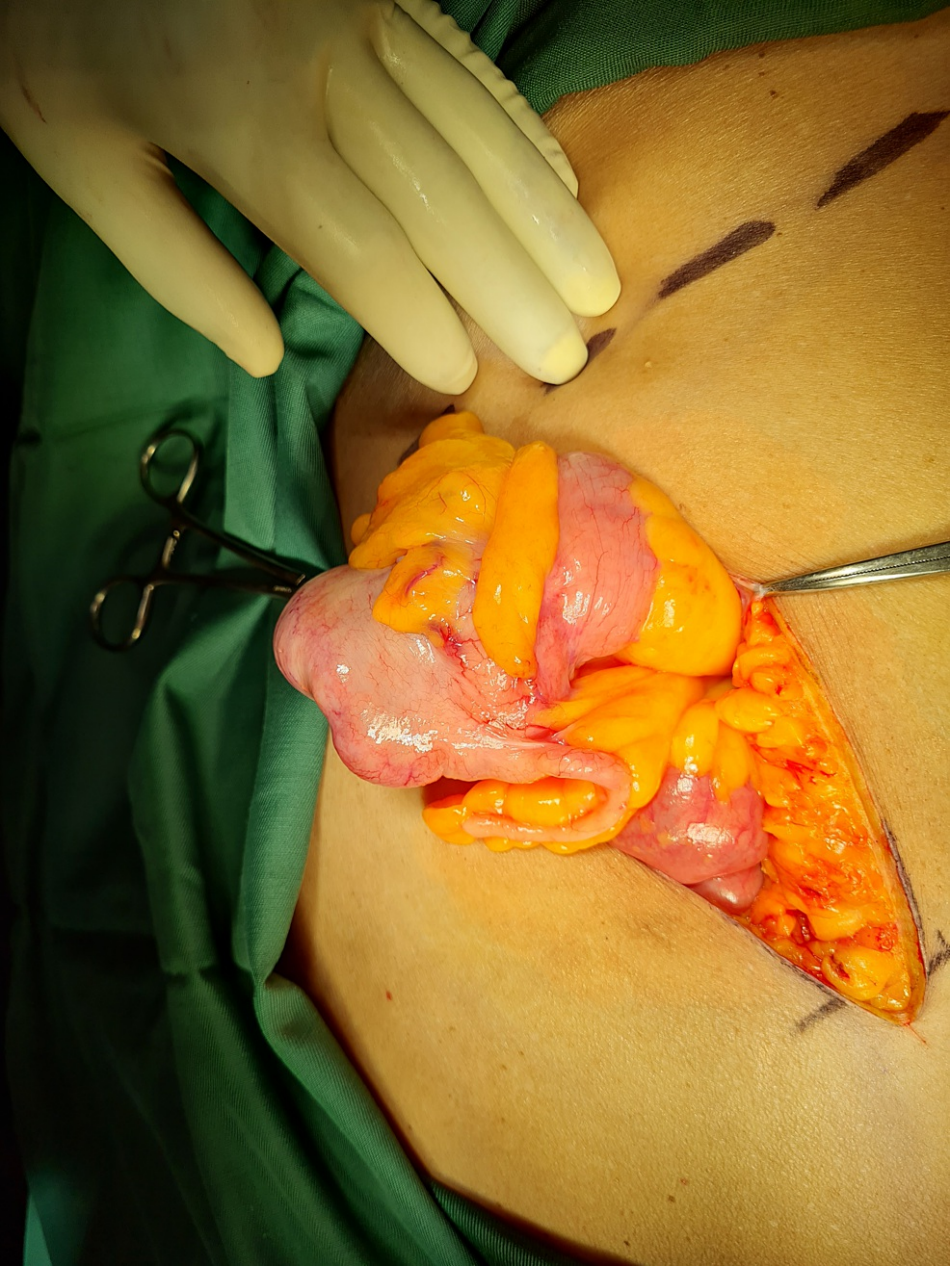
Right-sided Rutherford Morison incision

In view of the large defect present at her midline, the decision was made to perform the needed right hemicolectomy via a right-sided Rutherford Morison incision (Figures 5, 6).

**Figure 7:**
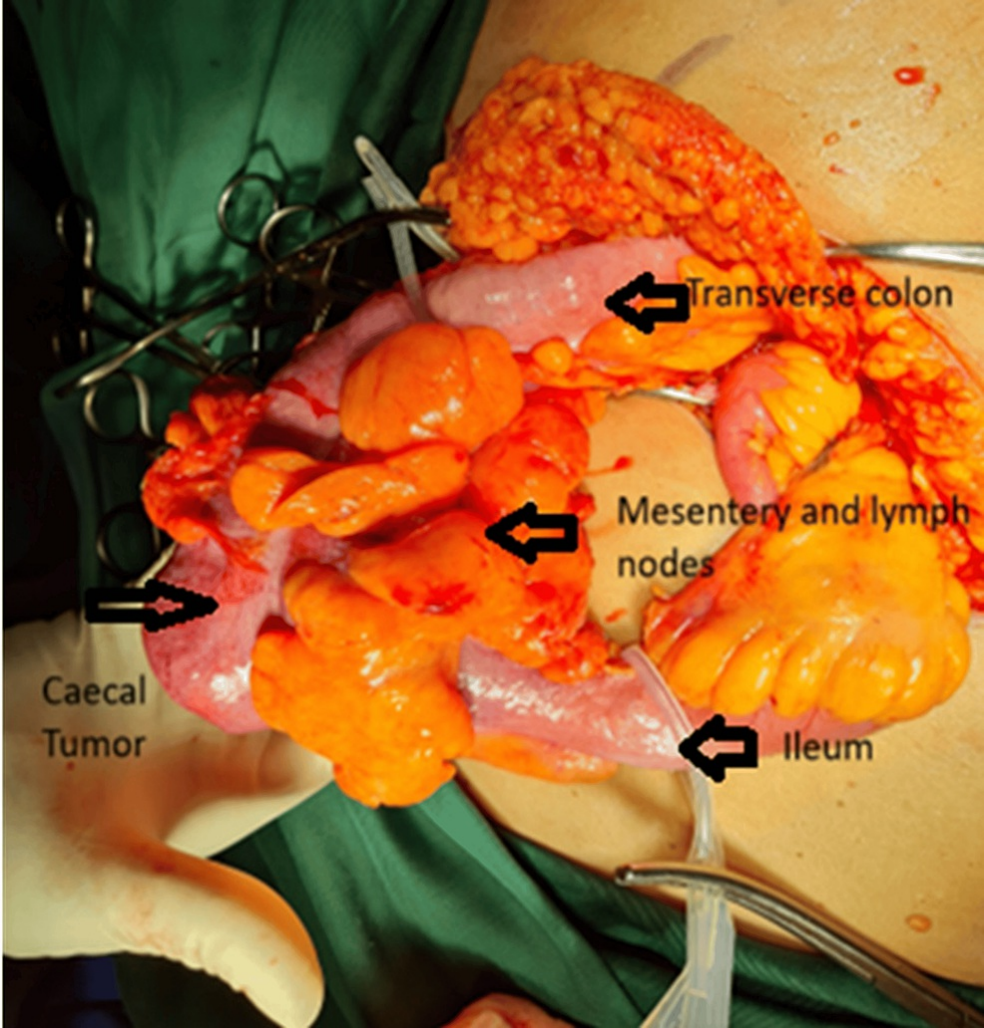
Intraoperative findings

**Figure 8:**
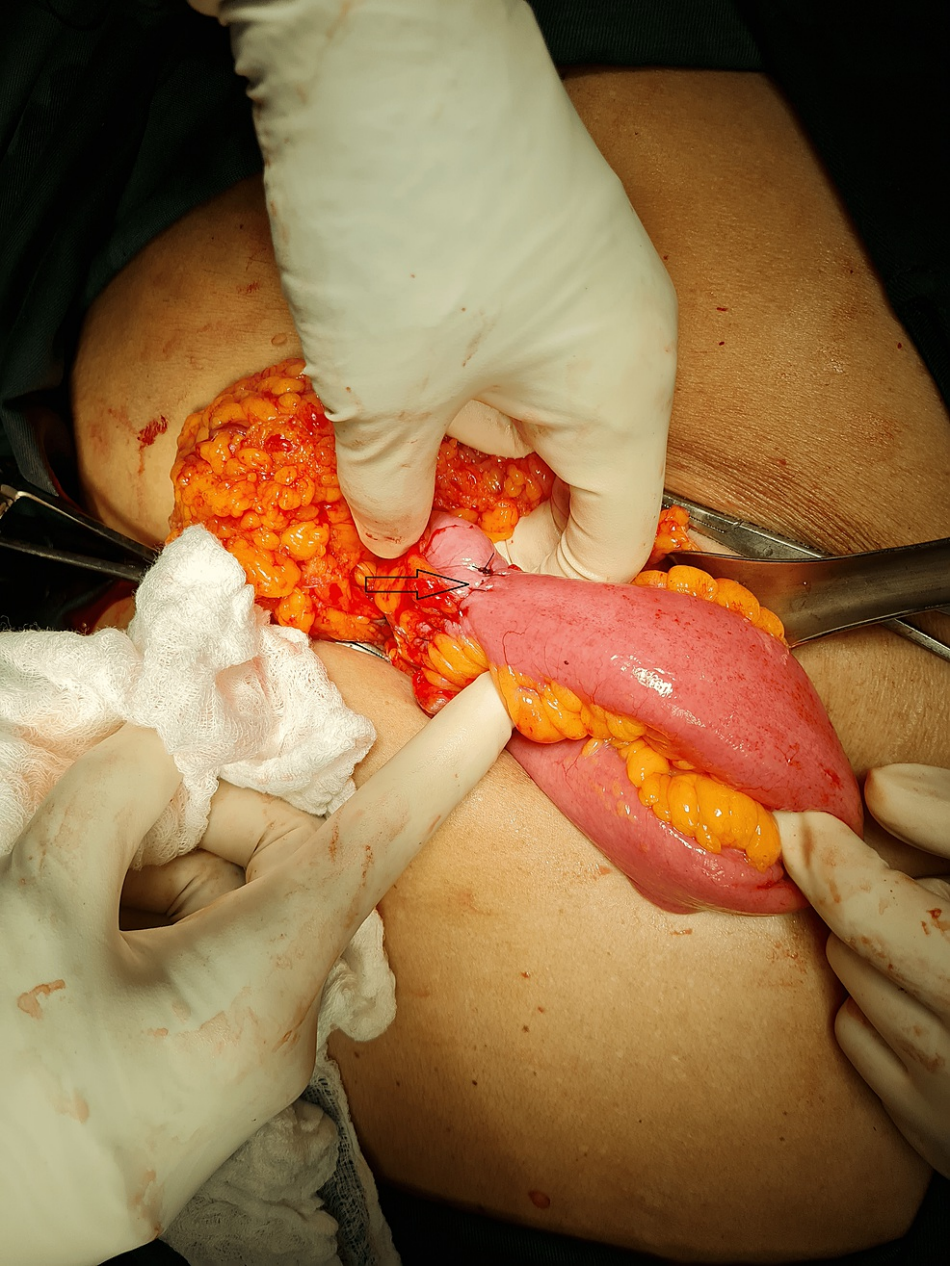
Hand-sewn end-to-end ileo-transverse anastomosis

**Figure 9:**
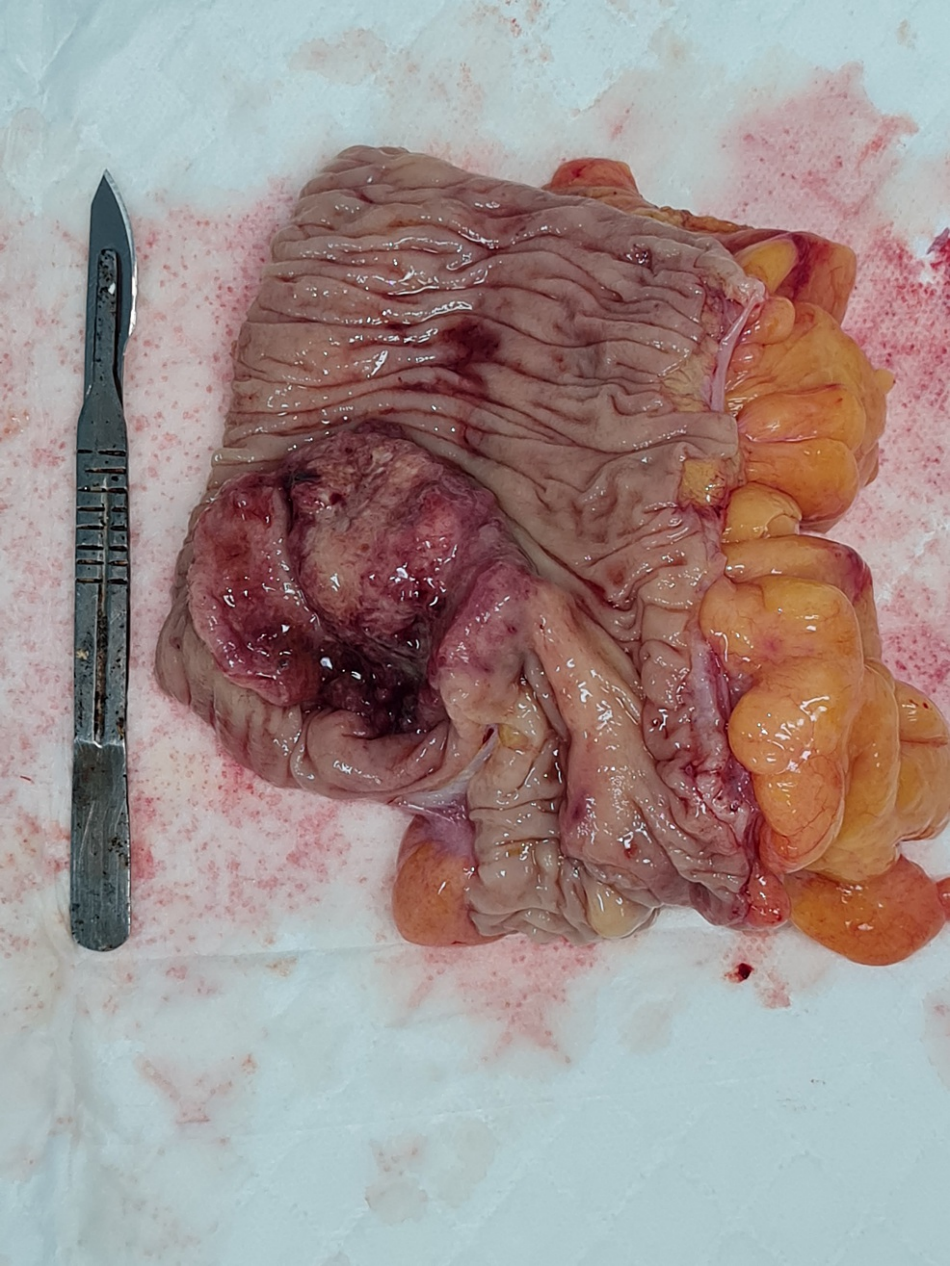
Resected specimen

**Figure 10:**
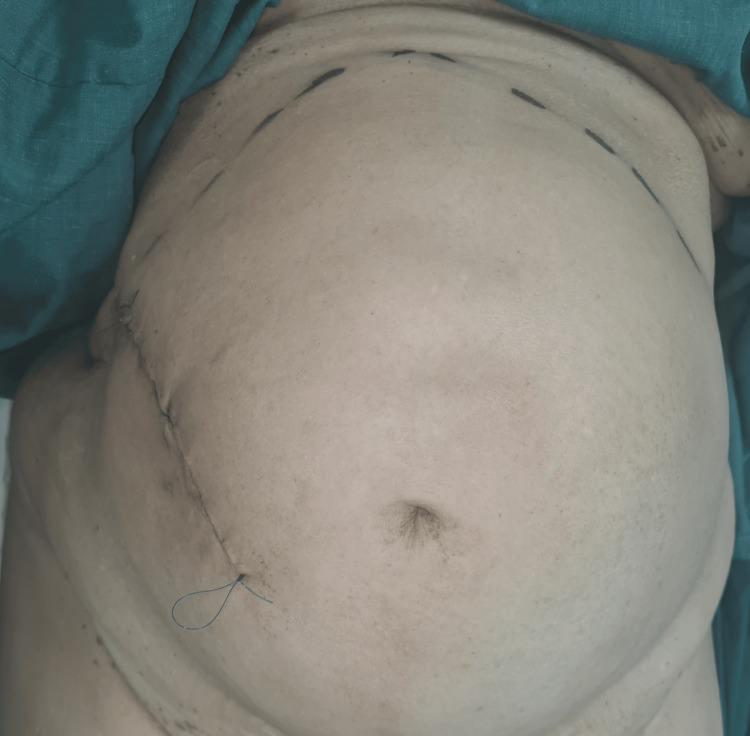
Closed Rutherford Morison incision

Surgical intervention (Figure 7) proceeded uneventfully with en-bloc resection of the tumour and retrieval of 15 lymph nodes. A hand-sewn, end-to-end, ileo-transverse anastomosis (Figure 8) was performed post-tumour resection (Figure 9). Histological evaluation confirmed adenocarcinoma of the caecum with two positive lymph nodes. Our patient went on to have adjuvant chemotherapy with an uncomplicated post-operative course (Figure 10).

## Discussion

Professor James Rutherford Morison was a renowned British surgeon, whose surgical practice spanned from the year 1888 to the early 1900s [3]. Professor Rutherford Morison made many invaluable contributions to the medical field, many of which are still in use today. Examples of such include the Rutherford Morison Tissue forceps, Bismuth Iodoform and Paraffin Paste, which he introduced during the First World War in the year 1916 [4,5], and most notably his description of the surgical anatomy of the right subhepatic space known as Morison’s pouch in 1894 [5,6].

In 1896 James Rutherford Morison pioneered the use of the Rutherford Morison incision, an oblique, curvilinear muscle-cutting incision. In his own writings, Rutherford Morison often described the incision as a long oblique incision in the left iliac region. In his more descriptive notations, he describes as follows, “An incision made in the left ileac fossa parallel to Poupart’s ligament and extending from the anterior superior spine to within 1 ½ inches of the symphysis pubis” [1].

Originally, this incision was performed on the left side, granting access to the sigmoid colon, and was particularly of use in cases where previous midline surgical intervention had resulted in midline scarring. Therefore, the Rutherford Morison incision allowed for easier intra-abdominal access when performing surgical procedures in the left lower quadrant.

This incision has been adapted for use in renal transplant surgery. Thus, its utilization is not limited to abdominopelvic access, but also extends to the retroperitoneum. While other incisions can be used, the Rutherford Morison predisposes to lower rates of incisional hernia formation as confirmed by Shahrestani et al. in a systematic review and meta-analysis which was focused on optimal surgical management in kidney transplantation, in order to minimize wound complications [7].

In the case described above, the presence of divarication of the rectus abdominis prompted the decision to implement the originally intended use of the Rutherford Morison incision. A search of the literature via MEDLINE, utilizing the keywords, ‘Rutherford Morison’ and ‘Rutherford Morison and hemicolectomy,’ did not reveal any documented cases utilizing this incision in the performance of hemicolectomy, other than in Professor Rutherford Morison’s writings [1]. Divarication of the rectus abdominis (DRAM), also known as diastasis recti (DR), is characterised by a thinned and widened linea alba accompanied by laxity of abdominal wall musculature. However, there is no true fascial defect present in this condition. Naraynsingh et al. re-emphasised the association between a weakened linea alba, termed, ‘Sick Linea Alba Complex (SLAC),’ with ventral hernia formation and concluded that once SLAC is excluded during closure of the abdominal wall, the incidence of herniation is significantly reduced or even eliminated [8]. The features of DR result in a midline bulge with increases in intra-abdominal pressure. In the case described, a midline incision was avoided, due to the risk of incisional hernia formation incurred by incision into an already weakened midline.

Unfortunately, despite advancements in techniques for abdominal wall closure, the rate of incisional hernia formation, following laparotomy is approximately 13-20%; with post-repair recurrence rates as high as 20-50% [9,10]. Complications accompanied by recurrence include pain and infection. When paired with DR, the rate of incisional hernia formation increases. This was demonstrated in a study conducted by Booth et al., who compared patients with DR to those without. They found the rate of incisional hernia formation in the presence and absence of DR to be 42% and 13% respectively [10]. In addition, the literature reveals an association between midline incisions performed in the presence of DR and burst abdomen [11].

With the current wave of minimally invasive procedures available, one may suggest the use of laparoscopy in the case described. Theoretically, laparoscopy can be used to avoid incision into a weakened midline in select cases. However, given the extent of the defect present within the abdominal wall of our index patient, port placement would have inevitably involved areas of weakness. Laparoscopy was deemed highly likely to result in port site hernia formation - a well-documented risk in DR [12,13]. Ki et al. performed a study revealing a trend toward port site hernia formation in cases of DR. Additional factors associated with port site herniation included increased body mass index, port site extension and sarcopenia, the latter having a 60% rate association with the presence of DR [13]. In laparoscopy, port site hernia prophylaxis often includes mesh, which we thought would increase the risk of post-operative sepsis in this case.

Of note, when undertaking a Rutherford Morison incisional approach, the operating surgeon must be wary of the traversing ilioinguinal and iliohypogastric nerves, entrapment to which can result in chronic pain [14].

## Conclusions

In summary, we have presented a case of a right hemicolectomy via the use of a right-sided Rutherford Morison incision, in a patient with concomitant diastasis of the recti. The use of this incision proves advantageous in cases necessitating intra-abdominal access, where a midline approach is not preferred. Its routine use in renal transplantation, in which patients are immunosuppressed, establishes it as a safe choice even in suboptimal cases. We therefore advocate for the reintroduction of the Rutherford Morison incision into routine surgical practice, as its usefulness in general surgery has been re-established, as in the days of James Rutherford Morison’s practice.
